# The vertebrate phylotypic stage and an early bilaterian-related stage in mouse embryogenesis defined by genomic information

**DOI:** 10.1186/1741-7007-5-1

**Published:** 2007-01-12

**Authors:** Naoki Irie, Atsuko Sehara-Fujisawa

**Affiliations:** 1 Department of Growth Regulation, Institute for Frontier Medical Sciences, Kyoto University, Kawahara-cho 53, Shogo-in, Kyoto 606-8507, Japan

## Abstract

**Background:**

Embryos of taxonomically different vertebrates are thought to pass through a stage in which they resemble one another morphologically. This "vertebrate phylotypic stage" may represent the basic vertebrate body plan that was established in the common ancestor of vertebrates. However, much controversy remains about when the phylotypic stage appears, and whether it even exists. To overcome the limitations of studies based on morphological comparison, we explored a comprehensive quantitative method for defining the constrained stage using expressed sequence tag (EST) data, gene ontologies (GO), and available genomes of various animals. If strong developmental constraints occur during the phylotypic stage of vertebrate embryos, then genes conserved among vertebrates would be highly expressed at this stage.

**Results:**

We established a novel method for evaluating the ancestral nature of mouse embryonic stages that does not depend on comparative morphology. The numerical "ancestor index" revealed that the mouse indeed has a highly conserved embryonic period at embryonic day 8.0–8.5, the time of appearance of the pharyngeal arch and somites. During this period, the mouse prominently expresses GO-determined developmental genes shared among vertebrates. Similar analyses revealed the existence of a bilaterian-related period, during which GO-determined developmental genes shared among bilaterians are markedly expressed at the cleavage-to-gastrulation period. The genes associated with the phylotypic stage identified by our method are essential in embryogenesis.

**Conclusion:**

Our results demonstrate that the mid-embryonic stage of the mouse is indeed highly constrained, supporting the existence of the phylotypic stage. Furthermore, this candidate stage is preceded by a putative bilaterian ancestor-related period. These results not only support the developmental hourglass model, but also highlight the hierarchical aspect of embryogenesis proposed by von Baer. Identification of conserved stages and tissues by this method in various animals would be a powerful tool to examine the phylotypic stage hypothesis, and to understand which kinds of developmental events and gene sets are evolutionarily constrained and how they limit the possible variations of animal basic body plans.

## Background

Comparative embryology of various animal groups has suggested the idea of a conserved stage in embryogenesis. In 1828, Karl von Baer was the first to describe revolutionary laws for animal development [1], inferring that the later the stage of embryogenesis of related organisms, the less they resemble each other [1,2]. This "progressive divergence model" was challenged by Seidel, Sander, and Elinson [3-5], who noted that earlier stages of development (such as patterns of cleavage) have relatively divergent morphological patterns among species, and so do not always fit the rule. Today, the revised version of the laws, called the "hourglass model" [6] or the "egg-timer model" [7], is widely accepted [4,8], and the bottleneck stage, representing maximum resemblance among various vertebrates, is commonly referred to as the "phylotypic stage" [9]. However, much controversy remains about when the phylotypic stage appears; Ballard first proposed the pharyngula stage [10] as the phylotypic stage, whereas Wolpert proposed the early somite segmentation stage [11], and Slack, the tailbud stage [12]. Moreover, a few detailed morphological studies have thrown doubt on the existence of a phylotypic stage [13,14]. These problems are due mainly to the difficulty in evaluating conserved or constrained developmental stages by morphological resemblance. In particular, heterochrony, or variations in the relative rate or timing of developmental events (e.g. pharyngeal arch formation and somitogenesis start almost at the same time in the mouse, but somitogenesis begins earlier in zebrafish), makes it extremely difficult to conclude which embryonic stage is the vertebrate phylotypic stage [15]. The problem of heterochrony prevents us from concluding whether each vertebrate really has a phylotypic stage, and whether any morphological features are associated with the stage.

Recent progress in developmental biology has revealed marked similarities of the molecular mechanisms of morphogenesis in bilaterians, leading to the "zootype" hypothesis [12]. The zootype is defined by a common pattern of Hox gene expression among bilaterians, and is considered to be displayed most clearly at the phylotypic stage. We note, however, many conserved genes other than Hox are also involved in animal development, so comprehensive evaluation of these genes should be included for a more accurate definition of zootype. Further, the fact that they are expressed differently during development among bilaterians prevents us from concluding which stage is zootype-related constrained. Recently, using mouse and human genome information, Hazkani-Covo et al. evaluated the protein distances of each gene expressed during mouse embryogenesis to show the existence of the vertebrate phylotypic stage [16]. Although they could not specify the timing of the phylotypic stage, they raised the possibility of a genome-based approach to examining the phylotypic stage hypothesis. Here, we sought to develop a novel method to evaluate the evolutionary ancestral nature of each embryonic stage comprehensively and quantitatively, without the use of comparative morphology but instead by evaluating the expression of conserved genes. By using this method, we tackled two questions. First, we asked whether the mouse embryo really passes through a highly constrained stage that is in accord with the vertebrate phylotypic stage hypothesis. Second, we asked whether the hourglass model could be expanded to cover the embryogenesis of vertebrates as members of the bilaterians, in other words, whether vertebrates pass through stages conserved among bilaterians.

## Results and discussion

### Estimating the ancestral nature of mouse embryogenesis

**Figure 1:**
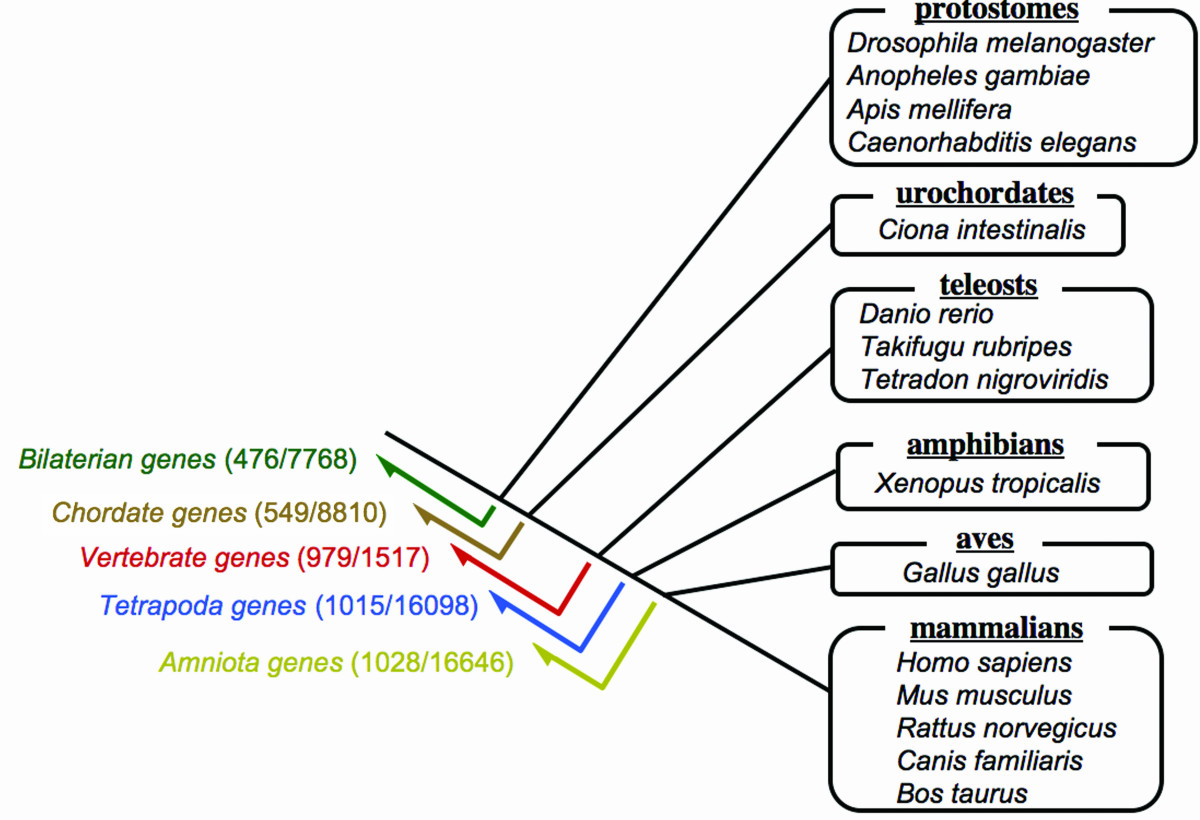
**Genome datasets used to define the evolutional classifications**. The evolutional classifications defined in our analyses are indicated, together with arrows covering the range of their putative evolutional origin (e.g. *Vertebrate genes* represent the genes that are considered to have already existed before vertebrate evolution). Numbers in parentheses indicate developmental genes and total genes classified in each evolutional classification. These classifications are consistent with a recent report [25, 26] that most of the duplications of developmental genes occurred before bilaterian and vertebrate evolution, supporting the appropriateness of our homologue determination (See Additional file 1: Taxonomic classification of homologues of mouse protein-coding genes according to taxonomic range.). See also Methods for the definition of evolutional classifications and "developmental genes".

On the basis that the vertebrate phylotypic stage should express the highest ratio of genes that conceivably existed in ancestral vertebrates, or *Vertebrate genes *(see Methods and Figure 1 for the definition of *Vertebrate genes*, also see Additional file 1: Taxonomic classification of homologues of mouse protein-coding genes according to taxonomic range.), we established the following index:

Vertebrate ancestor index at developmental stage k = V_k_/N_k_

Where V_k_: number of non-redundant *Vertebrate genes *expressed at stage k; N_k_: number of non-redundant total genes expressed at stage k.

Similarly, analyses with *Bilaterian*, *Chordate*, *Tetrapod*, and *Amniote genes* would also reveal the nature of each embryonic stage in a taxonomic context (see Methods for conserved gene definition).

To analyze the ancestor indexes of mouse embryonic stages, we collected mouse gene sets from the ENSEMBL database [17] and gene expression profiles from the Expressed Sequence Tags Database (dbEST) [18], and considered a gene to be "expressed" if the corresponding UniGene [19] ID was present in any of the categorized EST libraries. EST libraries categorized to each stage are listed in Table 1. Additionally, to make this analysis tolerant to the fluctuation in timing of embryonic stages, we took advantage of moving group analysis (similar to the well known "moving average analysis" technique) to calculate the ancestor index of two sequential stages:

Vertebrate ancestor index at grouped stage k = V_k_, _k+1_/N_k_, _k+1_

Where V_k, k+1_: number of non-redundant *Vertebrate genes *expressed at stage k or k+1; N_k_, _k+1_: number of non-redundant total genes expressed at stage k or k+1.

**Figure 2:**
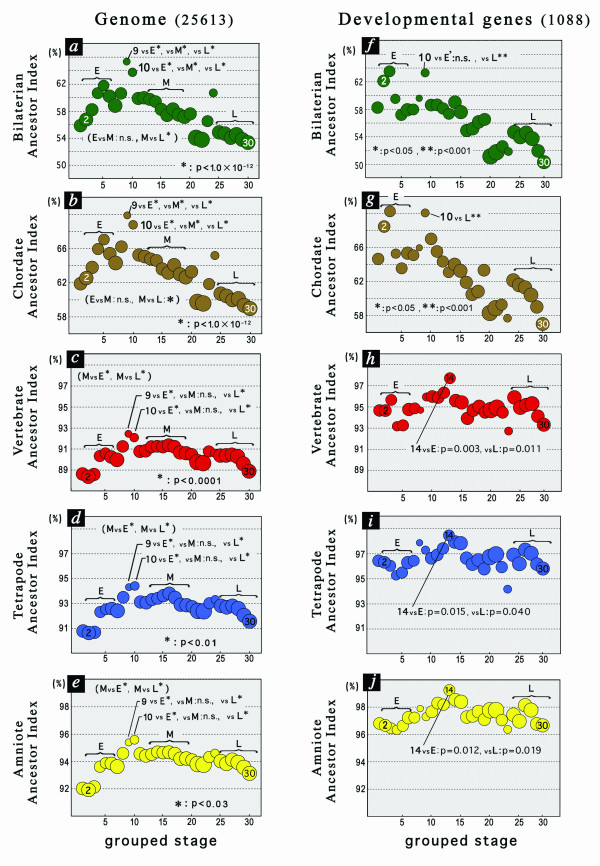
**Moving group analyses of individual ancestor indexes obtained during embryogenesis**. A–E: moving group analysis of the ancestor indexes within all genes (25,613 genes). Grouped stage number represents the earlier stage of a pair of developmental stages (e.g. gene expression of grouped stage 14 represents non-redundant genes expressed at stage 14 or 15; see Table 1 for staging). Each stage contained on average 22,744 EST counts (4,418–44,210), which corresponded to on average 4,396 (1,493–6,507) non-redundant ENSEMBL *M. musculus *genes. F–G: Moving group analysis of the ancestor indexes within developmental genes. Each stage contained on average 1,479 EST counts (282–3,406), which corresponded to 303 (94–491) non-redundant ENSEMBL *M. musculus *genes. E, M and L indicate the early, middle and late embryonic periods, covering non-redundant genes expressed in stages 2–7, 11–18 and 25–31, respectively. Statistical analysis was performed with Fisher's exact test. Significant differences are indicated by asterisks. n.s: P > 0.05. Within each graph, relative areas of circles reflect the number of expressed (total or developmental) genes at each stage.

With these methods, we calculated the bilaterian, chordate, vertebrate, tetrapod, and amniote ancestor indexes (Figure 2A–E).

What surprised us initially was the appearance of an early peak around grouped stages 4 to 10 from the analyses of *Bilaterian *and *Chordate genes* (Figure 2A,B). Broadly evaluated analysis also supported this tendency; both the early (stages 2–7) and middle embryonic periods (stages 11–18) have significantly higher ancestor indexes than the late period (stages 25–31). The prominently higher ancestor indexes shown in Figure 2A,B indicate the existence of novel, well-conserved stages conceivably carrying characteristics of the bilaterians at around the cleavage to blastula stages in mouse embryogenesis.

### Vertebrate phylotypic stage in mouse embryogenesis

We next calculated ancestor indexes to search for a putative phylotypic stage in mouse embryogenesis. In contrast to the bilaterian and chordate ancestor indexes, vertebrate, tetrapod, and amniote ancestor indexes did not show prominent peaks (Figure 2C–E. Instead, we obtained weakly curving graphs peaking around grouped stages 8 to 18 (Figure 2C–E), covering the presumptive periods of the vertebrate phylotypic stage proposed by comparative morphological studies (such as the pharyngula stage [10], the early somite segmentation stage [11], and the tailbud stage [12]). Importantly, the vertebrate ancestor index of the middle embryonic period was significantly higher than those of both the early and late embryonic periods (Figure 2C), as also seen in the tetrapod and amniote ancestor indexes (Figure 2D,E). All of these curves (Figure 2C–E) were significantly different from those obtained and shown in Figure 2A,B (P < 0.001; see Methods for the evaluation of ancestor index distribution).

If we were to see the body plan and morphological patterning as readouts of genetic programs for development, then the ancestor indexes calculated for developmental genes would refine our understanding of the conservation of the stages between 8 and 18. On this assumption, we focused on mouse developmental genes defined in the Gene Ontology database (see Methods for the definition of developmental genes) to derive ancestor indexes of individual stages. The highest ancestor index occurred at grouped stage 14 (stages 14–15), and was significantly higher than values of both the early and late embryonic periods (Figure 2H–J). Similar peaks in vertebrate, tetrapod, and amniote ancestor indexes indicate that E8.0–8.5 has been the stage most conserved throughout the evolution of vertebrates. Additionally, the peak of the amniote ancestor index at grouped stage 14 (Figure 2J) indicates that these stages express the smallest ratio of newest developmental genes. In light of the recent report that newer genes tend to evolve faster than older genes [20], the E8.0–8.5 period is the most genetically unmodifiable or contains the strongest developmental constraints in mouse embryogenesis. Thus, our analyses with developmental genes reveal that mouse embryogenesis indeed has a highly conserved stage characteristic of ancestral vertebrates, conceivably at around E8.0 to E8.5 (the period of onset of pharyngeal arch formation and somitogenesis), which is in accord with the concepts of a vertebrate phylotypic stage [6]. Notably, many of the mice with mutations in *vertebrate developmental genes* expressed at the putative vertebrate phylotypic stage have fatal or systemic phenotypes: embryonic or perinatal lethal phenotypes (52%), and abnormal phenotypes of the nervous (33%), craniofacial (14%), cardiovascular (24%), respiratory (12%), or skeletal (18%) system (see Additional file 2: Mutant phenotypes of vertebrate developmental genes expressed at the phylotypic stage). Thus, many of these gene products could be critical factors for defining fundamental morphological features of embryos. This information in turn implies that mutations of developmental genes expressed at the putative phylotypic stage are not easily accommodated, which also supports the idea that this stage tends to be evolutionarily conserved.

### Existence of bilaterian-related stage

Bilaterian and chordate ancestor indexes calculated with developmental genes again showed patterns significantly different from those of *vertebrate genes* (Figure 2F,G vs 2H–J; P < 0.001), with their highest values at grouped stages 3 and 10. Similar to the results obtained with genome-level analysis, both of these peaks were earlier than the putative vertebrate phylotypic stage, spanning from the period of cleavage to the onset of gastrulation (Table 1). These results suggest that this early embryonic period carries the basic body plan of bilaterians (i.e. bilaterally symmetric), making it the "bilateriotypic" stage. Gene expression information of *Ciona intestinalis *embryogenesis further allowed us to verify the existence of the putative bilaterian-related stage in this organism at the early embryonic stage (see Additional file 3: Ancestor indexes obtained for genes expressed during *Ciona intestinalis *embryogenesis). The highest value of bilaterian ancestor indexes of genes expressed during *C. intestinalis *embryogenesis occurred at the cleavage stage, and was significantly higher than the ancestor index of the later embryonic stage (combined expression data of larval and juvenile stages). However, we could not obtain evidence at the level of "developmental genes" owing to the scarcity of gene ontologies associated with *C. intestinalis *genes.

## Conclusion

We have developed a comprehensive quantitative method to evaluate evolutionarily constrained stages during the development of individual embryos. Our results show that mouse embryogenesis indeed passes through a highly constrained stage, as the hourglass model implies, at the mid-embryonic period (E8.0–8.5). They also show that the stage may be the most developmentally conserved stage since the evolution of ancestral vertebrates, making it a candidate for the phylotypic stage. Notably, it exhibits the major characteristics of the phylotypic stage proposed by morphological studies: pharyngula stage [10], early somite segmentation stage [11], and tailbud stage [12]. At the pharyngula stage, animals should contain a series of paired branchial grooves, a notochord, a post-anal tail, and a dorsal hollow nerve cord. In mice, maxillary components of the 1st branchial arch become prominent at about E8.0. The 3rd branchial arch is still absent when mandibular components appear at around E8.5. The first somite pairs appear at E8.0, and 8 to 13 somite pairs are found at E8.5. The tailbud stage is E9.5–10.5, later than the pharyngula and early somite segmentation stages. Thus, the period of E8.0–8.5 corresponds to the onset of the pharyngula stage and the early somite segmentation stage in mice. However, the exact characteristics of the phylotypic stage cannot be concluded from our results, as it should be common to all vertebrate embryos. Our method will be a powerful tool for comparison among diverse vertebrates. In contrast to morphological studies that evaluate developmental constraints qualitatively by morphological resemblance among embryos, our genome-based method evaluates developmental constraints quantitatively in a single species. This latter point is especially important to the testing of the phylotypic stage hypothesis: our method allows us to define constrained or conserved stages in each organism, and thus to compare data among various vertebrate embryos. This approach would clarify the common developmental events and gene sets that characterize the phylotypic stage (e.g., whether or not somitogenesis is dissociated [21] from the stage).

In addition, our results suggest the existence of a bilateriotypic stage within the cleavage to gastrulation period in mouse embryogenesis, and the possible conservation of this stage *in C. intestinalis*. These results demonstrate that although the early developmental period (e.g., cleavage) is relatively modifiable on an evolutionary time-scale compared with the phylotypic stage, the cleavage-to-gastrulation period still retains highly ancestral characteristics. On the basis of this perspective, we propose that mouse embryogenesis possesses two distinct, nested hourglasses, the vertebrate and bilaterian ones. These hourglasses may represent two major developmental constraints from distinct evolutional pasts – ancestors of vertebrates and bilaterians – in mouse embryogenesis. This extended version of the developmental hourglass model once again highlights the questioned, hierarchical nature of embryogenesis proposed by von Baer, albeit partly: "General features common to a large group of animals appear earlier in the embryo than do specialized features." Similar and further analyses in various animals will be required to verify this model, to explain how the bilateriotypic period defined by our analysis is related to the "zootype" hypothesis proposed by Slack [12], and to clarify certain aspects of the cleavage period shared among bilaterians [22]. Comprehensive gene expression data of various animals (e.g. fishes, birds, amphibians, insects, roundworms, and outgroup animals) and further development of gene ontology (GO) databases would make this kind of analysis more effective and definitive.

Defining conserved stages and extracting genes by our method would be a powerful tool to answer what kind of developmental events and gene sets are evolutionarily constrained. The information in turn would help explain how the conserved stages limit the possible variations of animal body plan in the context of animal evolution.

## Methods

### Genome and homologous gene dataset

Genome and homologue information for each gene were taken from ENSEMBL database [17] (v37, February 2006) using the data mining system BioMart [23]. Only "protein coding" gene types were used in our analysis (total of 25,613 ENSMUSG IDs in the *Mus musculus *genome; 14,278 ENSCING IDs in the *C. intestinalis *genome). Homologues represent reciprocal BLAST hits (including UBRH: unique best reciprocal hits; MBRH: multiple best reciprocal hits; and RHS: reciprocal hits based on synteny information) with an expectation (E)-value < 1e-10 (WU BLASTP, BLOSUM62). The genome data sets were versions NCBI m34 for *M. musculus *and JGI 2 for *C. intestinalis*. Taking gene loss during evolution into consideration [24], we defined the following evolutional classifications, which depend on the presence or absence of at least one homologue in a taxon of interest (see also Figure 1). *Bilaterian genes*: at least one homologue present in protostomes. *Chordate genes*: in addition to genes classified in *Bilaterian genes*, genes that have at least one homologue in urochordates. *Vertebrate genes*: in addition to genes classified in *Chordate genes*, genes that have at least one homologue in teleosts. *Tetrapod genes*: in addition to genes classified in *Vertebrate genes*, genes that have at least one homologue in amphibians. *Amniote genes*: in addition to genes classified in *Tetrapod genes*, genes that have at least one homologue in aves.

### Expression profile dataset

All the stage-traceable EST libraries of *M. musculus *and *C. intestinalis *were manually downloaded from the NCBI UniGene database [19] and categorized by their developmental stage descriptions (Table 1). We extracted all UniGene IDs with at least one entry in each categorized EST library, and further linked them to the ENSEMBL gene IDs. ENSEMBL genes with at least one of these UniGene IDs were considered as "expressed".

### Statistical analysis

For comparing ancestor indexes between stages, we constructed a 2 × 2 contingency table and determined significant differences between the ratios (e.g. non-redundant *Vertebrate gene *numbers expressed at stages k or k+1 and non-redundant total genes expressed at stages k or k+1) by Fisher's exact test (two-tailed). To test the distribution pattern of ancestor indexes, we converted the number of expressed *Bilaterian*, *Chordate*, *Vertebrate*, *Tetrapod*, and *Amniote genes* in each grouped stage to cumulative frequency distribution, and compared these survival curves (Kaplan-Meier method) by the Generalized Wilcoxon test. For all the statistical tests, P-values less than 0.05 were considered significant.

## Authors' contributions

NI conceived of and designed the study, and participated in acquiring and analyzing the data and drafting the manuscript. AS participated in designing the study and revising the manuscript critically for important intellectual content. Both authors have approved the final manuscript.

## Supplementary Material

Additional File 1 **Taxonomic classification of homologues of mouse protein-coding genes according to taxonomic range**. A: Taxonomic classification of mouse genome. B: Taxonomic classification of mouse developmental genes (see Methods for the definition of developmental genes). Classifications were defined by mouse homologues found in no other organisms (*M. musculus *only), those shared with *R. norvegicus *(Rodent); with *Homo sapiens*, *Canis familiaris *or *Bos taurus *(Mammal, but not Rodent); with *Gallus gallus *(Aves, but not Mammal); with *Xenopus tropicalis *(Amphibians, but not Aves); with *Danio rerio*, *Takifugu rubripes *or *Tetraodon nigroviridis *(Teleosts, but not Amphibians); with *Ciona intestinalis *(Ciona, but not Teleosts); with *Drosophila melanogaster*, *Anopheles gambiae, Apis mellifera *or *Caenorhabditis elegans *(Protostomes, but not Ciona); or with *Saccharomyces cerevisiae *(Yeast, but not Protostomes). Click here for file

Additional File 2 **Mutant phenotypes of *vertebrate developmental *genes expressed at the phylotypic stage**. Within 254 vertebrate developmental genes expressed at stages 14 or 15 (see Table 1 for staging), 159 ENSEMBL genes were associated with mutant mouse information at Mouse Genome Informatics [29]. The number of ENSEMBL genes linked to each Mammalian Phenotype ID is shown. MP, mammalian phenotype Click here for file

Additional File 3 **Ancestor indexes obtained for genes expressed during *Ciona intestinalis *embryogenesis**. A: EST libraries used to evaluate the ancestor indexes of genes expressed during *C. intestinalis *embryogenesis. Each stage contained on average 58,171 EST counts (43,912–98,568), which corresponded to 2,571 (2,156–3,098) non-redundant ENSEMBL *C. intestinalis *genes. B: Bilaterian ancestor indexes calculated for *C. intestinalis *embryogenesis (14,278 genes). *Bilaterian genes *were defined by at least one Ciona homologue present in protostomes (see Fig. 1 for genome datasets). L: non-redundant gene expression profile of late stages (larval and young adult). EST, expressed sequence tag; dbEST, database EST; E, embryonic day. Click here for file

## Figures and Tables

**Table 1:** EST library IDs categorized by the description of developmental stages. Each categorized mouse developmental stage contained about 22,300 (409–71,504) EST counts on average, which corresponded to 2,900 (194–5,923) non-redundant ENSEMBL gene entries on average. Developmental status was based on *Manipulating the Mouse Embryo*) [27] and *The Mouse *[28].

**Stage**	**Developmental status**	**dbEST library ID**
1	Unfertilized egg	16178, 16177, 10029, 14142, 1389, 867
2	Fertilized egg, 1 cell	12233, 2589, 12241, 1119, 17903
3	2 Cell	862, 1382, 5391
4	4 Cell	12243, 15709, 1524, 7328
5	8 Cell	9845, 15708, 1381
6	Morula	12244, 1532, 18029
7	Blastocyst, E4.0	12245, 10026, 1310, 1021, 875, 850, 18027, 12246, 18021
8	Trophoblast, ES cell	10033, 10023, 14556, 915, 10025, 10024, 14558
9	Formation of egg cylinder (E5.0)	12231, 18022
10	Proamniotic cavity in primitive ectoderm (E6.0)	2565
11	Advanced endometrial reaction, gastrulation starts, embryonic axis determined (E6.5)	13851, 10561
12	Primitive streak, amnion forming (E7.0)	5422, 547, 2381, 375, 2790, 12316
13	Neural plate, presomite stage (E7.5)	10036, 3777, 10562, 844
14	Neural folds, otic placode, 1–7 somites, hindgut pocket, 1st pharyngeal arch (E8.0)	2566, 1913
15	Neural folds close, 8–12 somites, 1st and 2nd pharyngeal arch, turning of embryo (E8.5)	10039, 538, 4042
16	Formation and closure of anterior neuropore, olfactory placode, 13–20 somites (E9.0)	5432, 7256
17	Forelimb bud, 21–29 somites (E9.5)	5392, 13850
18	Hindlimb and talbud, 30–34 somites (E10)	1884, 2517, 16788
19	Deep lens pit, 35–39 somites (E10.5)	1891, 15706, 528
20	Closure of lens vesicle, 40–44 somites (E11.0)	1885, 4142, 12312, 2557, 2578, 9944, 18018
21	Forefoot plate, lens vesicle detached (E11.5)	15707, 845
22	Hind foot plate, earliest sign of fingers (E12.0)	5319, 9846, 5464, 5423, 2514
23	Anterior footplate indented, (E13.0)	12258, 2521, 2554, 2521, 5469, 2555
24	Skin layers, chondrification of ribs (E13.5)	532, 15702
25	Fingers separate distally (E14.0)	12259, 2525
26	Fingers & toes separate (E15.0)	17914, 2596, 12265, 2522, 17951, 2594
27	Rib ossification (E15.5)	529, 12979
28	Reposition of umbilical hernia (E16.0)	12260, 2523, 18085, 12247
29	Wrinkled skin, umbilical hernia gone (E17.0)	12261, 18071, 12250, 12248, 12251, 2516, 1907
30	Auditory ossicles (E17.5)	12267, 18087
31	Iris and ciliary body (E18.0)	12266, 1770, 12313

## References

[B1] von Baer KE (1828). Entwicklungsgeschichte der Thiere: Beobachtung und Reflexion.

[B2] Hall BK (1992). Evolutionary Developmental Biology.

[B3] Seidel F (1960). Körpergrundgestalt und Keimstruktur eine Erörterung über die Grundlagen der vergleichenden und experimentellen Embryologie und deren Gültigkeit bei phylogenetischen Überlegungen. Zool Anz.

[B4] Sander K, Schmidt-Ott U (2004). Evo-devo aspects of classical and molecular data in a historical perspective. J Exp Zoolog B Mol Dev Evol.

[B5] Elinson RP, Raff RA, Raff EC (1987). Change in developmental patterns: embryos of amphibians with large eggs. Development as an Evolutionary Process.

[B6] Raff RA (1996). The Shape of Life: Genes, Development, and the Evolution of Animal Form.

[B7] Duboule D (1994). Temporal colinearity and the phylotypicprogression: a basis for the stability of a vertebrate Bauplan andthe evolution of morphologies through heterochrony. Dev Suppl.

[B8] Hall B (1997). Phylotypic stage or phantom: is there a highly conserved embryonic stage in vertebrates?. Trends Ecol Evol.

[B9] Sander K, Goodwin BC, Holder N, Wylie CC (1983). The evolution of patterning mechanisms: gleanings from insect embryogenesis and spermatogenesis. Development and Evolution.

[B10] Ballard WW (1981). Morphogenetic movements and fate maps of vertebrates. Am Zool.

[B11] Wolpert L (1991). The Triumph of the Embryo.

[B12] Slack JM, Holland PW, Graham CF (1993). The zootype and the phylotypic stage. Nature.

[B13] Bininda-Emonds OR, Jeffery JE, Richardson MK (2003). Inverting the hourglass: quantitative evidence against the phylotypic stage in vertebrate development. Proc Biol Sci.

[B14] Richardson MK, Hanken J, Gooneratne ML, Pieau C, Raynaud A, Selwood L, Wright GM (1997). There is no highly conserved embryonic stage in the vertebrates: implications for current theories of evolution and development. Anat Embryol (Berl).

[B15] Richardson MK (1995). Heterochrony and the phylotypic period. Dev Biol.

[B16] Hazkani-Covo E, Wool D, Graur D (2005). In search of the vertebrate phylotypic stage: a molecular examination of the developmental hourglass model and von Baer's third law. J Exp Zoolog B Mol Dev Evol.

[B17] Birney E, Andrews D, Caccamo M, Chen Y, Clarke L, Coates G, Cox T, Cunningham F, Curwen V, Cutts T (2006). Ensembl 2006. Nucleic Acids Res.

[B18] Boguski MS, Lowe TM, Tolstoshev CM (1993). dbEST – database for "expressed sequence tags". Nat Genet.

[B19] Joan U, Pontius LW, Schuler GD (2003). UniGene: a Unified View of the Transcriptome.

[B20] Alba MM, Castresana J (2005). Inverse relationship between evolutionary rate and age of mammalian genes. Mol Biol Evol.

[B21] Richardson MK, Allen SP, Wright GM, Raynaud A, Hanken J (1998). Somite number and vertebrate evolution. Development.

[B22] O'Farrell PH, Stumpff J, Su TT (2004). Embryonic cleavage cycles: how is a mouse like a fly?. Curr Biol.

[B23] Ensembl BioMart. http://www.ensembl.org/Multi/martview.

[B24] Hughes AL, Friedman R (2004). Differential loss of ancestral gene families as a source of genomic divergence in animals. Proc Biol Sci.

[B25] Pires-daSilva A, Sommer RJ (2003). The evolution of signalling pathways in animal development. Nat Rev Genet.

[B26] Miyata T, Suga H (2001). Divergence pattern of animal gene families and relationship with the Cambrian explosion. Bioessays.

[B27] Hogan BBR, Costantini F, Lacy E (1994). Manipulating the Mouse Embryo.

[B28] Rugh R (1990). The Mouse: Its Reproduction and Development.

[B29] Mouse Genome Informatics. http://www.informatics.jax.org.

