# Supplementary material for: The vertebrate phylotypic stage and an early bilaterian-related stage in mouse embryogenesis defined by genomic information
Source: BMC Biol. 2007 Jan 12;5:1. doi: 10.1186/1741-7007-5-1 (PMC1797197; doi:10.1186/1741-7007-5-1)
Supplement: Additional File 1 — Taxonomic classification of homologues of mouse protein-coding genes according to taxonomic range. A: Taxonomic classification of mouse genome. B: Taxonomic classification of mouse developmental genes (see Methods for the definition of developmental genes). Classifications were defined by mouse homologues found in no other organisms (M. musculus only), those shared with R. norvegicus (Rodent); with Homo sapiens, Canis familiaris or Bos taurus (Mammal, but not Rodent); with Gallus gallus (Aves, but not Mammal); with Xenopus tropicalis (Amphibians, but not Aves); with Danio rerio, Takifugu rubripes or Tetraodon nigroviridis (Teleosts, but not Amphibians); with Ciona intestinalis (Ciona, but not Teleosts); with Drosophila melanogaster, Anopheles gambiae, Apis mellifera or Caenorhabditis elegans (Protostomes, but not Ciona); or with Saccharomyces cerevisiae (Yeast, but not Protostomes). [file 1741-7007-5-1-S1.pdf]

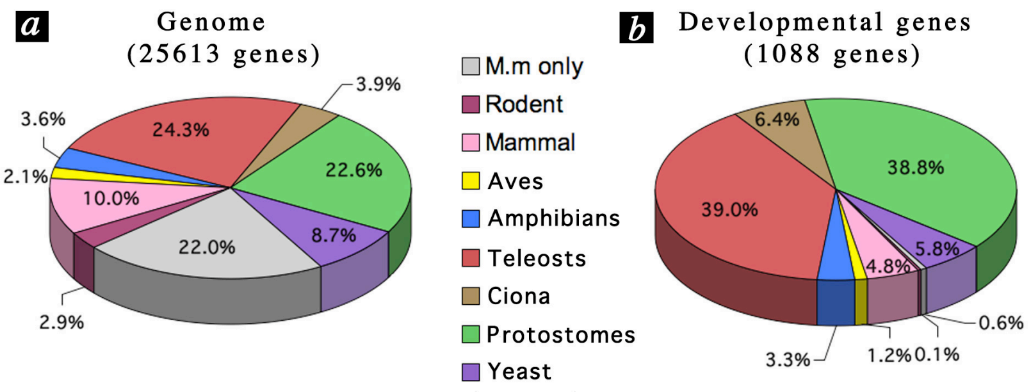

Additional file 1 - Taxonomic classification of homologues of mouse protein-coding genes according to taxonomic range.

a. Taxonomic classification of mouse genome **b.** Taxonomic classification of mouse developmental genes (see Methods for the definition of developmental genes). Classifications were defined by mouse homologues found in no other organisms (*M.m* only), those shared with *R. norvegicus* (Rodent); with *Homo sapiens*, *Canis familiaris* or *Bos taurus* (Mammal, but not Rodent); with *Gallus gallus* (Aves, but not Mammal); with *Xenopus tropicalis* (Amphibians, but not Aves); with *Danio rerio*, *Takifugu rubripes* or *Tetraodon nigroviridis* (Teleosts but not Amphibians); with *Ciona intestinalis* (Ciona but not Teleosts); with *Drosophila melanogaster*, *Anopheles gambiae, Apis mellifera* or *Caenorhabditis elegans* (Protostomes but not Ciona); or with *Saccharomyces cerevisiae* (Yeast but not Protostomes).
