# Supplementary material for: The vertebrate phylotypic stage and an early bilaterian-related stage in mouse embryogenesis defined by genomic information
Source: BMC Biol. 2007 Jan 12;5:1. doi: 10.1186/1741-7007-5-1 (PMC1797197; doi:10.1186/1741-7007-5-1)
Supplement: Additional File 2 — Mutant phenotypes of vertebrate developmental genes expressed at the phylotypic stage. Within 254 vertebrate developmental genes expressed at stages 14 or 15 (see Table 1 for staging), 159 ENSEMBL genes were associated with mutant mouse information at Mouse Genome Informatics [29]. The number of ENSEMBL genes linked to each Mammalian Phenotype ID is shown. MP, mammalian phenotype [file 1741-7007-5-1-S2.pdf]

number of genes linked to each MP ID	Mammalian Phenotype ID	Mammalian Phenotype description
5	MP:0001186	pigmentation phenotype
13	MP:0002006	tumorigenesis
14	MP:0002873	normal phenotype
2	MP:0003012	no phenotypic analysis
53	MP:0003631	nervous system phenotype
20	MP:0005367	renal/urinary system phenotype
18	MP:0005369	muscle phenotype
8	MP:0005370	liver/biliary system phenotype
22	MP:0005371	limbs/digits/tail phenotype
14	MP:0005372	life span-post-weaning/aging
22	MP:0005373	lethality-postnatal
82	MP:0005374	lethality-embryonic/perinatal
1	MP:0005375	adipose tissue phenotype
21	MP:0005376	homeostasis/metabolism phenotype
11	MP:0005377	hearing/ear phenotype
50	MP:0005378	growth/size phenotype
13	MP:0005379	endocrine/exocrine gland phenotype
52	MP:0005380	embryogenesis phenotype
16	MP:0005381	digestive/alimentary phenotype
22	MP:0005382	craniofacial phenotype
14	MP:0005384	cellular phenotype
38	MP:0005385	cardiovascular system phenotype
25	MP:0005386	behavior/neurological phenotype
31	MP:0005387	immune system phenotype
19	MP:0005388	respiratory system phenotype
20	MP:0005389	reproductive system phenotype
28	MP:0005390	skeleton phenotype
19	MP:0005391	vision/eye phenotype
2	MP:0005392	touch/vibrissae phenotype
8	MP:0005393	skin/coat/nails phenotype
3	MP:0005394	taste/olfaction phenotype
7	MP:0005395	other phenotype
24	MP:0005397	hematopoietic system phenotype

Within 254 Vertebrate-Developmental genes expressed at stages 14 or 15 (see Table 1 for staging), 159 ENSEMBL genes were associated with mutant mouse information at Mouse Genome Informatics [29]. The number of ENSEMBL genes linked to each Mammalian Phenotype ID is shown. MP, mammalian phenotype
