# Supplementary material for: The vertebrate phylotypic stage and an early bilaterian-related stage in mouse embryogenesis defined by genomic information
Source: BMC Biol. 2007 Jan 12;5:1. doi: 10.1186/1741-7007-5-1 (PMC1797197; doi:10.1186/1741-7007-5-1)
Supplement: Additional File 3 — Ancestor indexes obtained for genes expressed during Ciona intestinalis embryogenesis. A: EST libraries used to evaluate the ancestor indexes of genes expressed during C. intestinalis embryogenesis. Each stage contained on average 58,171 EST counts (43,912–98,568), which corresponded to 2,571 (2,156–3,098) non-redundant ENSEMBL C. intestinalis genes. B: Bilaterian ancestor indexes calculated for C. intestinalis embryogenesis (14,278 genes). Bilaterian genes were defined by at least one Ciona homologue present in protostomes (see Fig. 1 for genome datasets). L: non-redundant gene expression profile of late stages (larval and young adult). EST, expressed sequence tag; dbEST, database EST; E, embryonic day. [file 1741-7007-5-1-S3.pdf]

a *Ciona intestinalis*

Developmental status	*db EST library ID*
egg	9906
cleavage stage	9905, 11997
gastrula & neurula	12009
tailbud	9913
larva	9907, 10114
young adult (juvenile)	9904, 15834

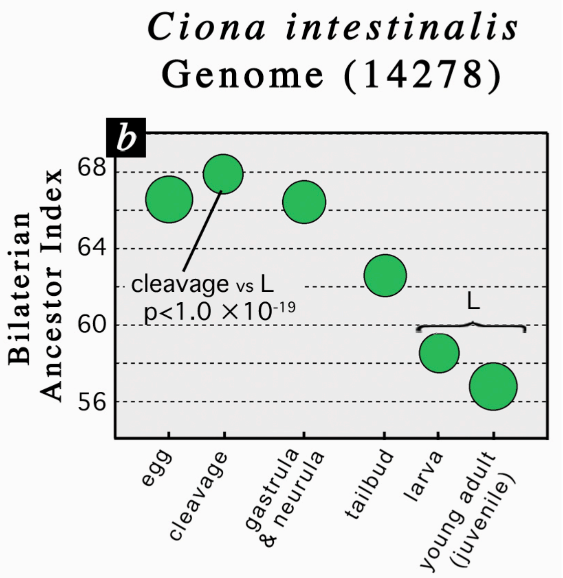

Supplementary Figure S2 Ancestor indexes obtained for genes expressed during *Ciona intestinalis* embryogenesis.

a. EST libraries used to evaluate the Ancestor Indexes of genes expressed during *C. intestinalis* embryogenesis. Each stage contained on average 58,171 EST counts (43,912–98,568), which corresponded to 2,571 (2,156–3,098) non-redundant ENSEMBL *C. intestinalis* genes. **b**, Bilaterian Ancestor Indexes calculated for *C. intestinalis* embryogenesis (14,278 genes). *Bilaterian genes* were defined by at least one *Ciona* homologue present in protostomes (see Fig. 1 for genome datasets). L: non-redundant gene expression profile of late stages (larval and young adult). EST, expressed sequence tag; dbEST, database EST; E, embryonic day.
